# Papayotin in Fissures of the Tongue

**Published:** 1887-06

**Authors:** 


### Papayotin in Fissures of the Tongue.

Professor Schwimmer reports excellent results from the use of papayotin in fissures of the tongue, after chromic acid, iodoform, and nitrate of silver had failed. The papayotin was administered in the following form:

℞. Papayotin..............................1 to 2 parts.
Distilled water......................
Glycerine............................ aa 10 "

This solution is to be applied with a camel's hair brush, from two to six times a day, the tongue having been previously well dried. There is no maceration, as would be supposed, but the mixture stops the pain and seems to cause a renewal of the epithelium. Schwimmer reports twenty-five cases, some of several years' duration; and complete cure was effected in all except one, which was syphilitic, though specific treatment gave us no good result. But in this case papayotin ameliorated the condition of the tongue.—*Wiener med. Wochenschrift.*

